# An Assessment of the Individual and Collective Effects of Variants on Height Using Twins and a Developmentally Informative Study Design

**DOI:** 10.1371/journal.pgen.1002413

**Published:** 2011-12-08

**Authors:** Scott I. Vrieze, Matt McGue, Michael B. Miller, Lisa N. Legrand, Nicholas J. Schork, William G. Iacono

**Affiliations:** 1 Psychology Department, University of Minnesota, Minneapolis, Minnesota, United States of America; 2 Minneapolis VA Medical Center, Minneapolis, Minnesota, United States of America; 3 The Scripps Translational Science Institute and the Scripps Research Institute, La Jolla, California, United States of America; Georgia Institute of Technology, United States of America

## Abstract

In a sample of 3,187 twins and 3,294 of their parents, we sought to investigate association of both individual variants and a genotype-based height score involving 176 of the 180 common genetic variants with adult height identified recently by the GIANT consortium. First, longitudinal observations on height spanning pre-adolescence through adulthood in the twin sample allowed us to investigate the separate effects of the previously identified SNPs on pre-pubertal height and pubertal growth spurt. We show that the effect of SNPs identified by the GIANT consortium is primarily on prepubertal height. Only one SNP, rs7759938 in *LIN28B*, approached a significant association with pubertal growth. Second, we show how using the twin data to control statistically for environmental variance can provide insight into the ultimate magnitude of SNP effects and consequently the genetic architecture of a phenotype. Specifically, we computed a genetic score by weighting SNPs according to their effects as assessed via meta-analysis. This weighted score accounted for 9.2% of the phenotypic variance in height, but 14.3% of the corresponding genetic variance. Longitudinal samples will be needed to understand the developmental context of common genetic variants identified through GWAS, while genetically informative designs will be helpful in accurately characterizing the extent to which these variants account for genetic, and not just phenotypic, variance.

## Author Summary

We evaluated the developmental specificity of 176 SNPs known to affect adult height based on meta-analysis from the GIANT consortium. First, longitudinal observations on height spanning pre-adolescence through adulthood in a twin sample allowed us to investigate the individual effects of the previously identified SNPs on both pre-pubertal height and pubertal growth spurt. We show that the effect of the SNPs identified by the GIANT consortium is primarily on prepubertal height. Only one SNP, rs7759938 in *LIN28B*, approached a significant association with pubertal growth. Second, using standard twin heritability models, we investigated the extent to which the collective effect of these SNPs explained genetic variance in height—as opposed to phenotypic variance, as other studies have done. We computed a genetic score by weighting SNPs according to their effects as assessed via meta-analysis. We show that, while the score accounts for ∼9% of the phenotypic variance in height (i.e., the overall variance), it accounts for ∼14% of the corresponding genetic variance. Longitudinal samples are necessary to understand the developmental context of common genetic variants identified through GWAS, while twin samples will be helpful in accurately characterizing the extent to which these variants account for genetic, and not just phenotypic, variance.

## Introduction

Adult height is a model multigenic phenotype for genetic association studies. Twin and adoption studies suggest that height is highly heritable (∼80%) [1], [2], [3], but the identification of individual genetic variants that contribute large effects to normal-range adult height (as with most complex traits) has proven to be very difficult [4], [5]. Despite this, it does appear possible that common SNPs with individually small effects can account for a large proportion of phenotypic variance in adult height (∼45%) [6] and may be identified with appropriately large sample sizes. To that end, the GIANT consortium has identified 180 SNPs that collectively account for 10.5% of variance in adult height in a sample of 183,727 individuals [7].

For any person adult height reflects roughly two decades of growth. Change in height is relatively rapid throughout infancy, slows down in early childhood, and increases again during puberty when a notable growth spurt occurs [8], [9]. The heritability of growth during any particular developmental period appears to be high, and it has been shown that some genetic variants affect a substantial proportion of height's phenotypic variance throughout development. For example, a longitudinal study of Swedish male twins found a genetic correlation of 0.73 between height at age 2 and at age 18, suggesting that 53% of the genetic variance in height at these ages is shared [10]. In contrast to this genetic consistency, the same study found that height measured during the pubertal growth spurt (ages 11 to 17) to be most influenced by new genetic variation. This differential effect of individual genetic variants on different stages of growth remains largely to be investigated, and the present study is a step in that direction. To accomplish this, we evaluated the relative effect of the SNP variants identified by [7] as part of the GIANT consortium efforts on both prepubertal height and growth during puberty.

## Materials and Methods

The sample (N = 6481) was drawn from Caucasian participants in the Minnesota Twin Family Study (MTFS). The MTFS a 20-year, longitudinal, community-representative study conducted at the University of Minnesota and approved by the University of Minnesota Institutional Review Board continuously since inception. The study has been extensively described previously [11]. The sample is composed of two cohorts of families. Twins in the younger cohort (N = 2046) were born between 1977 and 1994. They were initially evaluated at approximately age 11 (the youngest any individual was evaluated was at age 10.75). Twins in the older cohort (N = 1141) were born between the years 1972 and 1979 and were initially evaluated at approximately age 17. Both cohorts were followed-up at approximately three- to five-year intervals, with assessments thus targeting ages 11, 14, 17, 21, 24, and 29 years of age. Height was measured in a laboratory setting during visits to the University of Minnesota. Means and standard deviations for each twin's height and age of assessment at each wave are given in Table 1. Change in height is most rapid from age 11 to 14, with girls tapering off before boys, consistent with an earlier pubertal onset for girls [12]. Parents of the twins were also evaluated. There were 1332 fathers born between 1926 and 1976 with a mean height of 178.11 cm (SD = 6.51 cm). There were 1962 mothers born between 1934 and 1976 with a mean height of 165.03 cm (SD = 5.94 cm).

**Table 1:** Descriptive Statistics.

	Males					Females				
	N	MZ Pairs	DZ Pairs	Age (Mean SD)	Height in cm (Mean SD)	N	MZ Pairs	DZ Pairs	Age (Mean SD)	Height in cm (Mean SD)
Age 11	1016	327	168	11.78 (.40)	150.09 (7.80)	1012	315	179	11.76 (.45)	151.64 (7.68)
Age 14	880	281	146	14.85 (.48)	170.90 (8.19)	892	273	159	14.82 (.54)	163.79 (6.22)
Age 17	1170	371	196	17.71 (.51)	177.78 (6.74)	1320	425	218	17.75 (.59)	164.97 (6.32)
Age 20	916	287	142	21.31 (.86)	178.88 (6.65)	1126	348	195	20.76 (.56)	165.50 (6.35)
Age 24	817	242	130	24.72 (.96)	178.95 (6.61)	509	154	90	25.05 (.60)	166.42 (6.46)
Age 29	728	210	116	29.45 (.54)	179.17 (6.58)	566	178	85	29.49 (.51)	165.51 (6.17)

MZ is monozygotic twin; DZ is dizygotic.

The twin sample was used to develop a genetically mediated, longitudinal growth model for height. There are many approaches to analysis of longitudinal designs, and choosing the best approach often requires assumptions about, or explicit knowledge of, the (unknown) true growth trajectories generating the data [13]. Choosing a model for our sample was greatly facilitated by our ability to simultaneously leverage longitudinal measurements and twin zygosity information. We chose to model longitudinal height variability via a mixed model [14], because it could accommodate an intuitive variance-component decomposition to model growth through random intercept and random slope terms (i.e., the random effects). Covariates and SNPs were included in the model as fixed effects. We also accounted for twin zygosity by partitioning the random intercept and slope into additive genetic, shared environment, and unshared environmental effects using standard biometric twin methods [15]. Throughout the remainder of this paper we refer to this model simply as a ‘growth model.’

**Figure 1:**
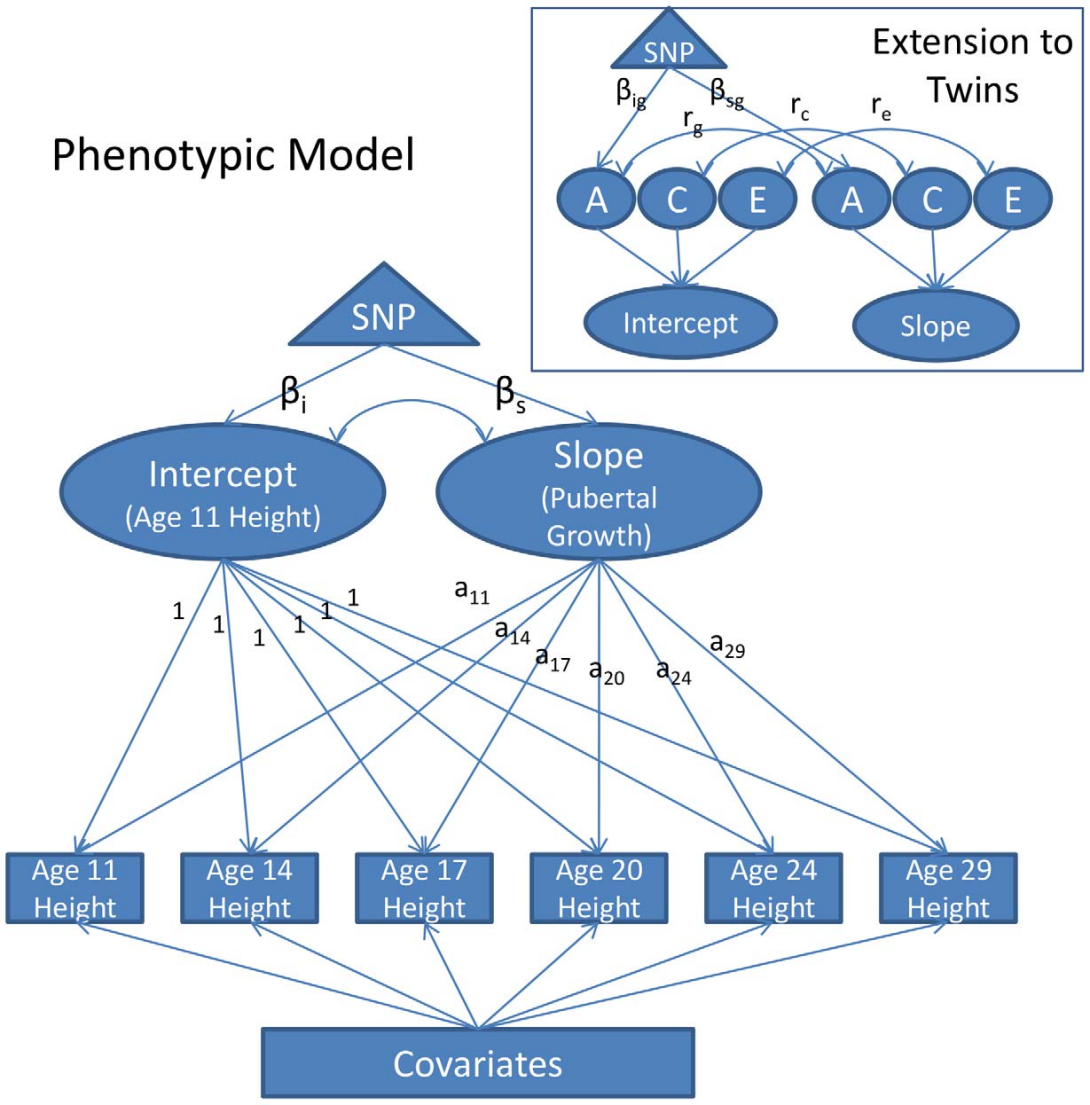
Diagram of the Piecewise Linear Growth Model. Measurements of height were taken at approximately ages 11, 14, 17, 20, 24, and 29. The measurements are adjusted for covariates, and then modeled as a function of a random intercept, representing age-11 height, and a random slope, representing growth in height from age 11 to adulthood, and a residual, which for convenience of presentation is not depicted in the figure. The intercept and the slope are allowed to correlate (this is represented as the double-headed arrow connecting them). The loadings of the slope onto height (denoted *a*_11_–*a*_29_) are the ages of participants during each of the assessments. The slope is piecewise linear in age, with the maximum age constrained to be 18 in males and 16 in males (see text for details). The effect of a SNP on the intercept and slope (denoted *β*_i_ and *β*_s_, respectively) can be estimated directly. The growth model extension to twins is portrayed in the inset box. By taking advantage of twin zygosity, the variance in the intercept and slope are partitioned into additive genetic (A), shared environmental (C), and unshared environmental (E) components. The correlation between the intercept and slope can also be decomposed in this way (e.g., *r*_g_ would be the genetic correlation, *r_c_* would be the shared environmental correlation, etc.). Using twins in this way, one can model the effect of a SNP onto the additive genetic variance in the intercept and slope, instead of merely the overall phenotypic variance. These effects are denoted as *β*_ig_ and *β*_sg_, respectively.

The growth model was constructed on the twins to evaluate SNP effects on age-11 height and pubertal growth after age 11. In the growth model, age-11 height corresponds to the intercept and pubertal growth corresponds to the slope. A diagram of the model is depicted in Figure 1. The full phenotypic diagram is portrayed. An extension to twins is included in the box inset.

In the path diagram of the phenotypic model (Figure 1) the observed measurements of height are represented by squares, one square for each height measurement taken (age 11, 14, 17, etc.). These measures are assumed to be a function of three random effects: one capturing variation in the intercept, one capturing variation in the slope, and one capturing variation in a residual effect. The intercept and slope variances were freely estimated. Covariate effects were treated as fixed effects. All height measures were centered at 10.75, the youngest age at which any participant was assessed (the mean age of the first assessment was 11.85). The intercept loads equally onto all height measurements, and is interpretable as height at (roughly) age 11. The slope reflects an individual's change in height from age 11 to adulthood. Loadings on the slope factor were fixed at each individual's actual age at assessment and so took into account the variation in age that existed at each assessment.

An important caveat with the proposed growth model as described in Figure 1 is its linear form. Growth in height is known to be nonlinear—growth velocity tapers during late adolescence and subsequently asymptotes. To account for this, without introducing a quadratic term, we used a piecewise linear approach. Consistent with previous literature [9], males were assumed to stop growing at any appreciable rate at age 18. Females were assumed to cease growing at age 16. These constraints were implemented by fixing all ages of assessment greater than 18 to be 18 in males, and constraining ages of assessment greater than 16 to be 16 in females.

As portrayed in Figure 1, the model allows one to test the effect of a SNP directly on the intercept (age 11 height) and slope (pubertal growth). This technique avoids multiple testing on each individual age of assessment (the square boxes) and carries all the advantages that come with the mixed model/variance components approach (e.g., full-information maximum likelihood estimation, increased precision in estimating the intercept and slope, etc. [14]).

Analogous to the phenotypic growth model is the twin growth model, partially displayed in the box inset in Figure 1. Here we see that the intercept and growth random effects can, with the use of twins, be further partitioned into three sources of variance: additive genetic variance (A), shared environmental variance (C), and unshared environmental variance (E). The SNP effect can then be constrained to affect only the genetic variance, effectively controlling for environmental noise in the phenotype.

With the proposed growth model, we could estimate the effect of SNPs on the intercept (i.e., height at age 10.75, the earliest age at which any participant was assessed) and slope (i.e., pubertal growth). Using twins, we could further estimate the influence of SNP effects on genetic variance—as opposed to overall phenotypic variance—thus providing a direct estimate of the genetic variance accounted for by the SNPs. Studies on height heretofore have been unable to accomplish this, and have been restricted to comparing estimates of the percent of variance accounted for by SNPs in their sample to estimates of heritability obtained in separate studies of twins (e.g., as in refs [6], [7], [16]).

There is considerable variation in age of pubertal onset. To obtain a more accurate measure of pubertal age (versus chronological age) we used the Pubertal Development Scale [17] for the age-11 and age-14 assessments. These measures were self-report items written to reflect Tanner stages, such as pubic hair and voice changes (in males), breast development, menarche, and skin changes (in girls). Each item is measured on a four-point scale and reliability/validity has been found to be acceptable [17]. For each sex, items were averaged to form overall puberty scores for each individual. These scores, and their correlations with height and age at the age-11 and age-14 assessments, are reported in Table 2. According to the mean differences and correlation patterns, the females are further along in pubertal self-ratings than boys at the age-11 assessment. The girl's puberty score is also more highly correlated with height during the age-11 assessment than the age-14 assessment, indicating an early pubertal growth spurt in girls. The reverse is true for males, their puberty score has a low correlation with height at age 11 and a higher correlation at age 14, indicating a later pubertal growth spurt. At the age-11 assessment 15% of females had experienced menarche, versus 93% by the age-14 assessment. Average age of menarche was 12.8 years (SD = 1.0)

**Table 2:** Measures of Pubertal Status.

Age of Assessment	Mean	Median	SD	Correlation with age at that assessment	Correlation with height at that assessment
Males					
Age-11 Puberty	1.3	1.3	0.49	0.21	0.28
Age-14 Puberty	2.7	2.8	0.58	0.35	0.47
Females					
Age-11 Puberty	2.2	2.3	0.64	0.28	0.51
Age-14 Puberty	3.3	3.3	0.44	0.28	0.21

Pubertal stage as measured by a self-report questionnaire inquiring about Tanner stages of pubic and body hair growth, voice changes in boys, and breast development and menarche in girls. Responses (except for menarche) are recorded on a four-point scale (1–4).

We incorporated pubertal status into the growth model in the following way. For each age of assessment we regressed height on age and puberty score and, for females, an indicator variable measuring whether menarche had occurred by that assessment. For males both age and puberty score were highly significant in predicting height at age 11 (r^2^ = .17, p<2e^−16^) and 14 (r^2^ = .23, p<2e^−16^). In females at age 11 age, puberty score, and menarche significantly predicted height (r^2^ = .32, p<2e^−16^). At age 14 only the puberty score was a significant predictor (r^2^ = .04, p = 8.4e^−9^). We computed the sum of age and puberty score (and menarche for females) weighted by their corresponding regression weight and scaled the result to have the mean and variance of the original chronological age at 11 and 14. This gives an estimate of each subject's pubertal age—as opposed to chronological age—as it relates to growth in height. In supplementary analyses these pubertal ages were used in place of chronological age for the age-11 and age-14 assessments in the growth model, in an attempt to more precisely gauge the developmental specificity of each SNP.

All statistical analysis was conducted in the R Environment [18]; growth models were fit via maximum likelihood with the OpenMx package [19]. R scripts that implement the models used in this paper are available upon request.

### Genotyping and Imputation

SNPs were genotyped on an Illumina 660quad array using DNA derived from whole blood for approximately 90% of the sample and from saliva samples for the remainder. For quality control purposes, each 96-well plate included DNA from two members of a single CEPH family (rotated across plates) and one duplicate sample. Markers were excluded if (see ref [20] for additional details): 1) they had been identified as a poorly genotyped marker by Illumina; 2) had more than one mismatch in duplicated QC samples; 3) had a call rate <99%; 4) had a MAF <1%; 5) had more than 2 Mendelian inconsistencies across families; 6) significantly deviated from Hardy-Weinberg equilibrium at p<1e-7; 7) was an autosomal marker but associated with sex at p<1e-7; 8) had a significant batch effect at p<1e-7; or 8) there were more than 2 heterozygous X chromosome calls for males or mitochondrial calls for anyone. In total, 32,153 (5.7%) of the 559,982 SNP markers were eliminated by these screens, with the majority (3.6%) being eliminated because of low MAF. Samples were eliminated if: 1) they had >5000 no-calls; 2) had a low GenCall score; 3) had extreme heterozygosity or homozygosity; or 4) represented a sample mix-up or we could not confirm known genetic relationships. In total, 160 (2.2%) of the total genotyped sample of 7438 failed one or more of these criteria, with the majority (1.7%) failing because of low call rate.

Of the 180 SNPs described by the GIANT consortium as associated with height, 52 existed on the Illumina 660quad. The remaining were imputed with best-guess genotypes using MaCH [21], [22] and haplotypes from the 1000 Genomes 2010-06 reference dataset. Of the 128 imputed SNPs, three had poor imputation quality (rs17511102, rs11144688, rs473902; r^2^ = .08, .46, and .20). One SNP (rs5017948) was not contained in the 1000 Genomes 2010-06 or 2010-08 reference datasets and so was discarded. The average r^2^ of the remaining 124 imputed SNPs was .96 (SD = .07, range = [.55, 1.0]). In total, 176 of the 180 SNPs from the GIANT Consortium meta-analysis [7] were available in the current dataset. A genetic score was created by summing these 176 SNPs, weighted by their individual meta-analytic regression coefficient reported in the GIANT Consortium report [7]. All analyses accounted for the following covariates: sex, year of birth, cohort status (younger versus older), and the 10 first principal components from Eigenstrat [23] based on a subsample of 10,000 SNPs from sample founders (i.e., unrelated subjects).

## Results

**Figure 2:**
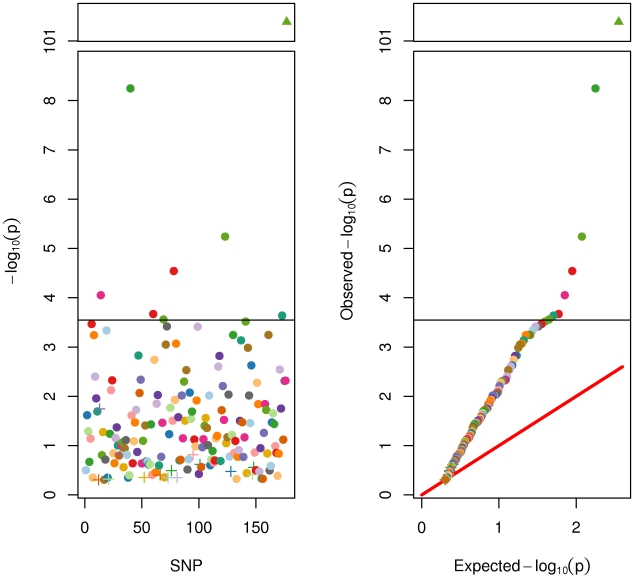
SNP and Genetic Score Effects for Adult Height. On the left panel are the univariate associations for each of the 176 SNPs (of the 180 SNPs from the Allen et al. [7] meta-analysis) ordered by chromosomal location along the x-axis. On the right is the same information portrayed in a QQ plot. . Note the discontinuous y-axis. Filled circles represent SNPs with direction of effect equal to that reported in the meta-analysis. “+” symbols represent SNPs with effects opposite to that reported in the meta-analysis. The filled triangle represents the genetic score. All *p*-values are also reported in Table S1. SNPs in Figure 2 and Figure 3 are colored to faciliatate cross-referencing between panels and between figures.

First, we ran association tests for each of the 176 SNPs on adult height in the full sample. A subset of twins (N = 775) had not yet reached adult height and were excluded from this analysis (i.e., were male and under 18 at the time of their last assessment, or were female and under 16). To account statistically for within-family clustering a generalized least squares method was used [24]. One-tailed test results are displayed in Figure 2. A few trends are clear. First, there was insufficient power in the present sample (N = 5706) to detect a genome-wide significant association for many SNPs. This was not surprising because 185,000 subjects were required in the GIANT Consortium [7] to reliably identify these SNPs. Six SNPs were significant at the Bonferroni correction for 176 tests at alpha of .05. Ninety-eight of the 170 SNPs were nominally statistically significant at .05. Despite a lack of genome-wide or Bonferroni significance, it was clear from the QQ plot that the vast majority of SNPs had lower p-values than expected by chance. This was clearly true for the genetic score based on the 176 SNPs, which was highly significant (p = 4e^−102^) and accounted for 9.2% (95% confidence interval = [8.2%, 11.1%]) of the phenotypic variance in adult height (in the full sample N = 5706). This result is not significantly different from the r^2^ of 10.5% found by the GIANT Consortium in [7]. As described in detail later, this same SNP score accounted for 14.3% (95% c.i. = [.3%, 26%]) of the additive genetic variance in adult height, as estimated in the twin sample alone.

Fitting the growth model, we found the intercept was 86% heritable (95% c.i. = [69%, 100%]) and the slope 84% heritable (65%, 100%). Shared environmental effects accounted for 9% (0%, 27%) and 11% (0%, 31%) of the variance in the intercept and slope, respectively. Unshared environment accounted for 5% (4%, 6%) in the intercept and 5% (4%, 7%) in the slope. Clearly, both age-11 height and pubertal growth (here, the intercept and slope) were highly heritable, consistent with previous reports [2]. The total phenotypic correlation between age-11 height and pubertal growth of −.62 indicates that taller individuals at age 11 experienced less growth after age 11. The genetic correlation between age-11 height and pubertal growth was −.56 (−.70, −.41), indicating that only 31% of genetic effects on age-11 height and pubertal growth are shared. Shared and non-shared environmental correlations were negligible (−.03 and −.03, respectively, both non-significant).

**Figure 3:**
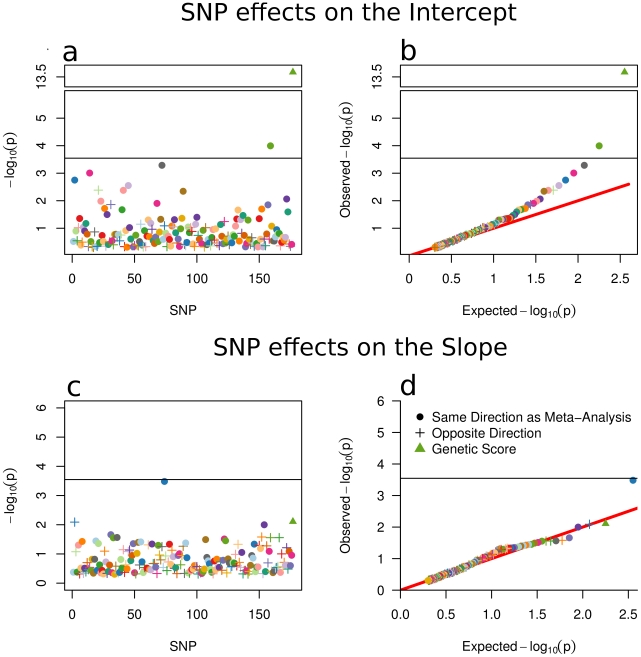
SNP and Score Effects on the Growth Model Intercept and Slope. Univariate plot (a) and QQ plot (b) of SNP effects on intercept. Univariate plot (c) and QQ plot (d) of SNP effects on the slope. All symbols are described in the caption for Figure 2. The SNP effects are relatively stronger for age-11 height (i.e., the intercept; panels (a) and (b)) than for pubertal growth (i.e., the slope; panels (c) and (d)). The genetic score is highly significant for age-11 height but not pubertal growth. The single Bonferroni-significant effect for the slope (highest blue dot in panels (c) and (d)) is the only SNP, of all 180 identified in the Allen et al. [7] meta-analysis, that has been linked to pubertal growth in height [26]. All *p*-values are also listed in Table S1. Each mark is colored to allow easy cross-referencing between panels, and also between Figure 2 and Figure 3.

Individual SNP and genetic score effects on the intercept and slope were computed simultaneously. Log-transformed p-values are displayed in Figure 3. Several trends are clear. First, only one p-value was significant based on a Bonferroni correction for 176 tests for the intercept (age-11 height). Only one SNP (rs7759938) approached significance for the slope. This extends previous findings for the relationship between rs7759938 and pubertal growth [25], [26]. Second, 49 (28%) SNP effects on the intercept and 77 (44%) SNP effects on the slope were in the opposite direction as reported in the meta-analysis. A binomial test of whether the proportion of SNPs in the opposite direction was smaller than that expected by chance for the intercept (*p* = 2e^−9^), but was chance-level for the slope (*p* = .06). The QQ plot for age-11 height clearly showed higher −log10(p) values than expected by chance, and the genetic score was highly significant (t = 7.58, df = 1652, *p*<1e^−13^). Alternatively, the QQ plot for pubertal growth consistently showed chance-level effects. The genetic score effect here was nominally significant (t = 2.42, df = 1652, *p*<.004).

All regression coefficients, standard errors, and *p*-values for the SNP effects on the growth model intercept, the growth model slope, and adult height are listed in Table S1. Table S2 contains the correlation matrix of regression coefficients from the meta-analysis [7], the SNP effect on the growth model's intercept and slope, and coefficients from the analysis of adult height presented earlier. The correlations of these regression coefficients were statistically significant, indicating that the general trend of SNP effects was similar across all height phenotypes. Coefficients from the adult height analysis were correlated most strongly with the meta-analytic coefficients (.81), followed by the SNP effects on the intercept (.46) and slope (.17). Figure S1 is a scatterplot matrix of these regression coefficients, illustrating the general trends of covariance among them.

**Figure 4:**
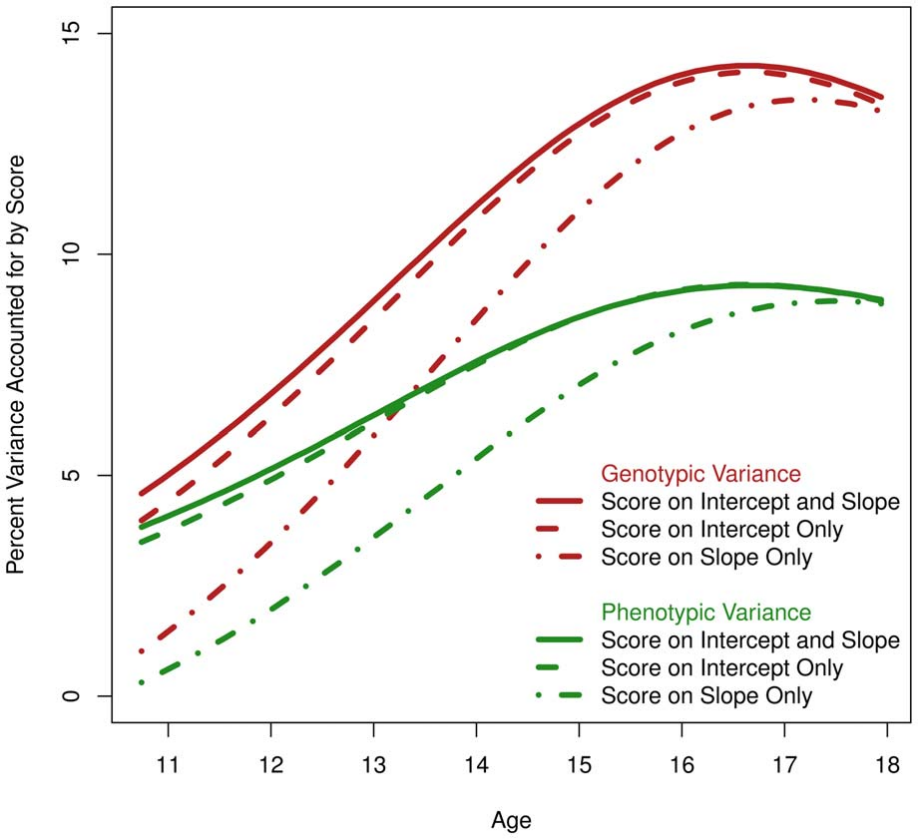
Phenotypic and Genetic Variance in Height Accounted for by Three Models (i.e., r^2^). Green indicates the r^2^ of the score effect on phenotypic variance in height (using the growth model represented in Figure 1). Red indicates the r^2^ of the score effect on genetic variance in height (using the growth model extension to twins displayed in the inset box of Figure 1). Clearly, focusing the score effect onto the twin model's additive genetic variance, and thereby controlling for environmental noise, allows larger estimates of r^2^. Irrespective of phenotypic or genetic variance, allowing the score to affect only the growth model slope resulted in the lowest r^2^ for both conditions. Allowing the score to affect only the growth model intercept resulted in considerable gain in r^2^. Most interestingly, allowing the score to simultaneously influence the slope and intercept resulted in negligible gain over allowing the score to influence the intercept only (i.e., the effect of score onto slope fixed at zero). This indicates that the score is only negligibly related to genetic variation specific to pubertal growth (slope), but rather is relevant to genetic variation that affects growth occurring up to age 11 (intercept). In addition, it provides evidence that some genetic variation indexed by the score is relevant for growth both before age 11 and from age 11 to adulthood.

To summarize findings from the growth model, we computed the variance in height accounted for by the genetic risk score's effect on the intercept and slope. A linear growth model is linear in the mean function but quadratic in the variance. In the present model, phenotypic height (*h*) is a quadratic function of the intercept (*i*), slope (*s*), and age (*a*),
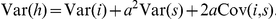



This parabola was computed for the base model, with no genetic score effect, and used to compute r^2^ with the genetic score. We plotted phenotypic r^2^ in Figure 4 in green for three models: 1) a model with a genetic score effect on the slope only, 2) a model with a score effect on the intercept only, and 3) the full model with a score effect simultaneously on intercept and slope. Taken together, differences between these models allowed estimation of the independent contribution of the genetic score on age-11 height versus pubertal growth. Apparent in Figure 4 was that the score is accounting for variance in height (all models including the score result in positive and significant r^2^). The model with a genetic score effect only on the slope did account for variance in height at all ages, but not as much as the model with a score effect only on the intercept. More to the point, the model with score effect simultaneously on the slope and intercept negligibly improved over the model with score effect only on the intercept. This indicated that the score has only a slight relationship with the unique SNP variance in pubertal growth. However, because the score reduces variance in height even when it was only allowed to load onto the slope, it did appear that the score is related to genetic variance overlapping between the intercept and slope (recall the intercept and slope are genetically correlated at −.56, and we expected some genetic variants to affect both).

Using the twins, we also computed the genetic r^2^, or the genetic variance in height accounted for by the genetic score. This is plotted in red in Figure 4 for the same three models described in the previous paragraph. As expected, the genetic r^2^ was greater than the phenotypic r^2^ for the entire age range under investigation. Comparing the maximum phenotypic r^2^ to the maximum genetic r^2^ for the model with a score effect on both the intercept and slope, one notices a jump from 9.2% (95% c.i. = [3.0%, 15.1%]) to 14.3% (95% c.i. = [0.3%, 26%]; each measured at the respective function's apex). That is, by using the twins to partition environmental variance, we were able to increase the magnitude of the SNP effect and consequently the sensitivity of the analysis.

### Sex-Specific Analysis

Growth models were also fit separately to the male and female subsamples. After scaling male heights for each age of assessment to have the female's mean and variance, the growth model variance component parameters were different between the sexes (χ^2^ = 78.04, *df* = 9, *p* = 4e^−13^). While heritability of the intercept was similar (.84 for males versus .82 for females) the heritability of the slope was different (.93 versus .64, respectively). Females had a larger shared environmental contribution to their slope variance (.01 for males and .31 for females). Males and females had similar phenotypic correlations between the intercept and slope (−.66 for males and −.64 for females). The genetic and environmental contributions to this correlation were different between the sexes. The genetic correlation between intercept and slope was −.60 for males and −.47 for females. The shared environment correlation was −.04 for males and −.14 for females. The unshared environmental correlation was −.02 in males and −.03 in females.

The overall SNP association trends were similar in both sexes (i.e. larger effects on the intercept and smaller effects on the slope). All SNP and score statistics are included in Table S1. Notable differences included the following. The effect of rs7759938 on pubertal growth is only significant for females (see Table S1). This sex difference has been noted previously [26]. Second, as can be seen in Figure S2, the overall genetic and phenotypic variance accounted for in height by the score is larger for males than for females.

### Incorporating Measures of Puberty into the Growth Model

Growth model parameters did not change dramatically after correcting chronological ages at 11 and 14 for pubertal status. The negative correlation between the intercept and slope was unchanged (−.62) with a larger genetic contribution (−.58) and smaller contributions by shared environment (−.02) and unshared environment (−.03). The intercept was 88% heritable with contributions of 7% and 5% from shared and unshared environment, respectively. The slope was 84% heritable with contributions of 10% and 6% from shared and unshared environment, respectively.

SNP associations also remained largely unchanged after correcting for pubertal status. Figure S3 gives association plots for the puberty-corrected associations. The correlation between regression weights from corrected versus uncorrected models was very high, for associations with the intercept (*r* = .99) and the slope (*r* = .99). The mean regression weight onto the intercept in the uncorrected model was .06 (SD = .43) versus .05 (SD = .43). The mean weight onto the slope in the uncorrected model was −.005 (SD = .06) versus −.003 (SD = .06). Correlations between standard errors and *p*-values were equally similar between the corrected and uncorrected models.

## Discussion

The first wave of GWAS research has been successful in identifying numerous common variants associated with various adult disorders and traits [27]. Yet virtually all disorders and traits are a consequence of a sequence of developmental processes, and we know very little about how these genetic variants play out across development. Research on *FTO*, where the minor allele of rs9939609 is a well established risk-factor for adult obesity, illustrates the importance of a developmental perspective. Specifically, the minor allele of rs9939609 is negatively associated with body mass index (BMI) until the age of 2.5, but, because it is associated with an earlier onset of the adiposity rebound that occurs in childhood, positively associated with BMI after age 5.5 years [28]. Research placing genetic association results in a developmental context will be necessary to understand how genetic variants contribute to a phenotype and, in the context of disease phenotypes and personalized medicine, to determine when and how intervention and/or prevention is possible.

The present study extended genetic analysis of developmental phenotypes by implementing a growth model to partition observed measures into two biologically meaningful constructs: pre-pubertal height and pubertal growth. We focused here on an established literature of SNP effects on height. This is necessary because individual genetic effects are too small to be detected at genome-wide levels by most individual studies, and combining longitudinal studies with commensurate phenotypes can be prohibitively difficult (longitudinal data is expensive and rare, investigators gathering different data on individuals from different populations at different ages and developmental levels). It may be that consortia of cross-sectional data will largely be necessary to discover replicable genetic variants while smaller, methodologically-unique individual studies will be left to understand those effects within a developmental context.

The vast majority of SNPs identified by Allen et al. [7] appear to be more strongly related to pre-pubertal height than to the pubertal growth spurt. The sample size precludes definite conclusions without replication or meta-analysis, however. In addition, age is only a fallible proxy for developmental stage or pubertal status. While many boys are expected to be pre-pubertal at age 10.75, this is less certain for females. In the present study 15% of females had already experienced menarche by the time they were first assessed. When we adjusted the ages for pubertal status, however, the results were highly similar to those using uncorrected ages. Nonetheless, future work evaluating genetic effects on growth would clearly benefit from including younger ages of assessment and more frequent follow up.

While most SNPs were unrelated to pubertal growth, one was. rs7759938 in *LIN28B* has previously been identified as relevant for adult height [7] and timing of pubertal onset [26], [29], [30]. Transgenic mice in ortholog *Lin28a* were found to have accelerated growth during the first 60 weeks of life in addition to later onset of puberty [25]. Our analysis also found accelerated growth related to the G allele of rs7759938. However, the effect was not significant for males and was confounded with pubertal onset for females, as about 15% of our 11-year-old females had already entered puberty by their age-11 assessment [31]. The effect remained in females even after adjustment for pubertal status, suggesting the variant is associated with rate of growth during these ages. The effect remained non-significant for males even when later growth periods were used as an attempt to better measure pubertal onset (i.e., investigating growth from age 14 to adulthood or age 17 to adulthood). The lack of an effect for males appears to be a sex-moderated effect (ref [26], [30] also reported small effects for males).

We also evaluated the effect of a genetic score on zygosity-derived genetic variance, as opposed to phenotypic variance, using a sample of twins. The score accounted for 14.3% of genetic variance in adult height, but only 9.2% of phenotypic variance, illustrating the possible advantages of using a twin sample. The use of twins provides concrete advantages over analyses that estimate the fraction of heritable variance attributable to multiple loci indirectly either based on previously reported heritability estimates or genome-wide markers in unrelated individuals. Admittedly, the advantage may not be extremely powerful in the present context, given height's high heritability, where the genetic variance is 80% or more of the total phenotypic variance. However, for less-heritable phenotypes, or where heritability is less well known, the approach will provide improved information about the magnitude of a SNP's (or gene's, or pathway's) relationship to the phenotype. A growth model is not necessary to evaluate genetic r^2^, but so-called “genetically informative” samples such as twins or adoptive families are. An array of statistical techniques have been developed for such samples [15], and incorporating genetic variants like SNPs is always possible and in many cases straightforward.

In summary, genomic findings from consortia may be fruitfully characterized within a developmental framework. Many analytic approaches exist, and the best may depend on the data structure at hand. Genetically informative samples such as twins remain important and viable tools in investigating genomic variation, even as genotyping or sequencing becomes routine.

## Supporting Information

Figure S1 Scatterplot Matrix of Regression Coefficients. “Meta-Analysis” refers to the SNPs reported in Allen et al. [7]. “Age-11” Height refers to the intercept in the growth model reported in text. “Pubertal Growth” refers to the slope of that growth model. “Adult Height” refers to regression coefficients from the adult height analysis.
(TIF)

Figure S2 (a) Refers to males; (b) to females. All other aspects are described in the caption to Figure 4 in the main text.
(TIF)

Figure S3 SNP and Score Effects on the Puberty-Corrected Growth Model Intercept and Slope. Univariate plot (a) and QQ plot (b) of SNP effects on intercept. Univariate plot (c) and QQ plot (d) of SNP effects on the slope. All symbols are described in the caption for Figure 2. The SNP effects are relatively stronger for age-11 height (panels (a) and (b)) than for pubertal growth (panels (c) and (d)). The genetic score is highly significant for age-11 height but not pubertal growth. The single Bonferroni-significant effect for the slope (highest blue dot in panels (c) and (d)) is the only SNP, of all 180 identified in the Allen et al. [7] meta-analysis, that has been linked to pubertal growth in height [26]. All p-values are also listed in Table S1. Each mark is colored to allow easy cross-referencing between panels, and also between Figure 2 and Figure 3.
(TIF)

Table S1 Gene/allele information, regression coefficients, standard errors, and p-values for all tests in the growth model and the adult height analysis.
(XLS)

Table S2 Regression Correlation Coefficient Matrix. These are Spearman rank order correlations between all 176 regression coefficients computed for each height phenotype under study. Confidence intervals were computed by bootstrap with 2000 pseudo-replications. “Meta-Analysis” refers to sex-combined regression coefficients from the GIANT Consortium meta-analysis [7]. “Age-11 Height” and “Pubertal Growth” are the SNP effects on the growth model intercept and slope, respectively. “Adult Height” is the full adult height analysis described in the present report. All values are statistically significant, indicating a general trend for the SNP effects regardless of height phenotype analyzed. The full scatterplot matrix is given in Figure S1.
(DOC)

## References

[1] Macgregor S, Cornes BK, Martin NG, Visscher PM (2006). Bias, precision and heritability of self-reported and clinically measured height in Australian twins.. Hum Genet.

[2] Silventoinen K, Sammalisto S, Perola M, Boomsma DI, Cornes BK (2003). Heritability of adult body height: A comparative study of twin cohorts in eight countries.. Twin Res.

[3] Visscher PM, Hill WG, Wray NR (2008). Heritability in the genomics era - concepts and misconceptions.. Nat Rev Genet.

[4] Maher B (2008). Personal genomes: The case of the missing heritability.. Nature.

[5] Manolio TA, Collins FS, Cox NJ, Goldstein DB, Hindorff LA (2009). Finding the missing heritability of complex diseases.. Nature.

[6] Yang JA, Benyamin B, McEvoy BP, Gordon S, Henders AK (2010). Common SNPs explain a large proportion of the heritability for human height.. Nature Genetics.

[7] Allen HL, Estrada K, Lettre G, Berndt SI, Weedon MN (2010). Hundreds of variants clustered in genomic loci and biological pathways affect human height.. Nature.

[8] Okasha M, Gunnell D, Holly J, Smith GD (2002). Childhood growth and adult cancer.. Best Pract Res Cl En.

[9] Tanner JM, Whitehouse RH, Takaishi M (1966). Standards from Birth to Maturity for Height, Weight, Height Velocity, and Weight Velocity: British Children, 1965. Part II.. Archives of Disease in Childhood.

[10] Silventoinen K, Pietilainen KH, Tynelius P, Sorensen TIA, Kaprio J (2008). Genetic regulation of growth from birth to 18 years of age: The Swedish young male twins study.. Am J Hum Biol.

[11] Iacono WG, McGue M (2002). Minnesota Twin Family Study.. Twin Res.

[12] Palmert MR, Boepple PA (2001). Variation in the timing of puberty: Clinical spectrum and genetic investigation.. J Clin Endocr Metab.

[13] Salem RM, O'Connor DT, Schork NJ (2010). Curve-based multivariate distance matrix regression analysis: application to genetic association analyses involving repeated measures.. Physiol Genomics.

[14] Pinheiro JC, Bates DM (2000). Mixed-effects models in S and S-PLUS.

[15] Neale MC, Cardon LR (1992). Methodology for genetic studies of twins and families.

[16] Visscher PM, Yang JA, Goddard ME (2010). A Commentary on ‘Common SNPs Explain a Large Proportion of the Heritability for Human Height’ by Yang et al. (2010).. Twin Research and Human Genetics.

[17] Petersen AC, Crockett L, Richards M, Boxer A (1988). A self-report measure of pubertal status: reliability, validity, and initial norms.. Journal of Youth and Adolescence.

[18] R Development Core Team (2011). R: A language and environment for statistical computing.

[19] Boker S, Neale M, Maes H, Wilde M, Spiegel M (2011). OpenMx: An Open Source Extended Structural Equation Modeling Framework.. Psychometrika.

[20] Miller MB, Basu S, Cunningham J, Oetting W, Schork NJ (submitted). The Minnesota Center for Twin and Family Research Genome-Wide Association Study..

[21] Li Y, Willer CJ, Ding J, Scheet P, Abecasis GR (2010). MaCH: Using Sequence and Genotype Data to Estimate Haplotypes and Unobserved Genotypes.. Genet Epidemiol.

[22] Li Y, Willer C, Sanna S, Abecasis G (2009). Genotype Imputation.. Annu Rev Genom Hum G.

[23] Price AL, Patterson NJ, Plenge RM, Weinblatt ME, Shadick NA (2006). Principal components analysis corrects for stratification in genome-wide association studies.. Nature Genetics.

[24] Li X, Basu S, Miller MB, Iacono WG, McGue M (2011). A Rapid Generalized Least Squares Model for a Genome-Wide Quantitative Trait Association Analysis in Families.. Hum Hered.

[25] Zhu H, Shah S, Shyh-Chang N, Shinoda G, Einhorn WS (2010). Lin28a transgenic mice manifest size and puberty phenotypes identified in human genetic association studies.. Nature Genetics.

[26] Widen E, Ripatti S, Cousminer DL, Surakka I, Lappalainen T (2010). Distinct Variants at LIN28B Influence Growth in Height from Birth to Adulthood.. Am J Hum Genet.

[27] Hindorff LA, Sethupathy P, Junkins HA, Ramos EM, Mehta JP (2009). Potential etiologic and functional implications of genome-wide association loci for human diseases and traits.. P Natl Acad Sci USA.

[28] Sovio U, Mook-Kanamori DO, Warrington NM, Lawrence R, Briollais L (2011). Association between Common Variation at the FTO Locus and Changes in Body Mass Index from Infancy to Late Childhood: The Complex Nature of Genetic Association through Growth and Development.. PLoS Genet.

[29] Uitterlinden AG, Perry JRB, Stolk L, Franceschini N, Lunetta KL (2009). Meta-analysis of genome-wide association data identifies two loci influencing age at menarche.. Nature Genetics.

[30] Ong KK, Elks CE, Li SX, Zhao JH, Luan J (2009). Genetic variation in LIN28B is associated with the timing of puberty.. Nature Genetics.

[31] Burt SA, McGue M, Demarte JA, Krueger RF, Iacono WG (2006). Timing of menarche and the origins of conduct disorder.. Arch Gen Psychiat.

