# Supplementary figures and images for: An Assessment of the Individual and Collective Effects of Variants on Height Using Twins and a Developmentally Informative Study Design
Source: PLoS Genet. 2011 Dec 8;7(12):e1002413. doi: 10.1371/journal.pgen.1002413 (PMC3234218; doi:10.1371/journal.pgen.1002413)

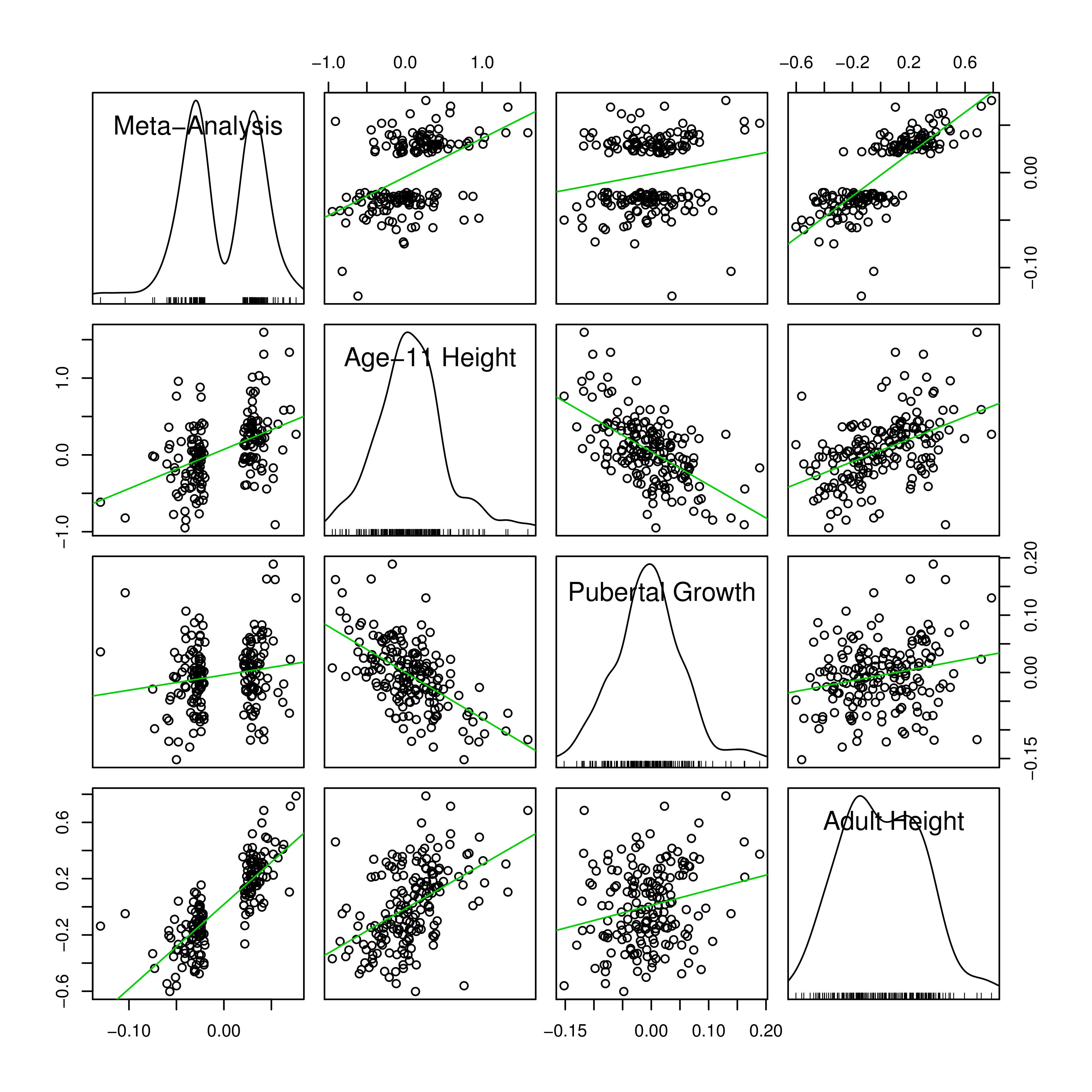

Supplement: Figure S1 — Scatterplot Matrix of Regression Coefficients. “Meta-Analysis” refers to the SNPs reported in Allen et al. [7]. “Age-11” Height refers to the intercept in the growth model reported in text. “Pubertal Growth” refers to the slope of that growth model. “Adult Height” refers to regression coefficients from the adult height analysis. (TIF) [file pgen.1002413.s001.tif]

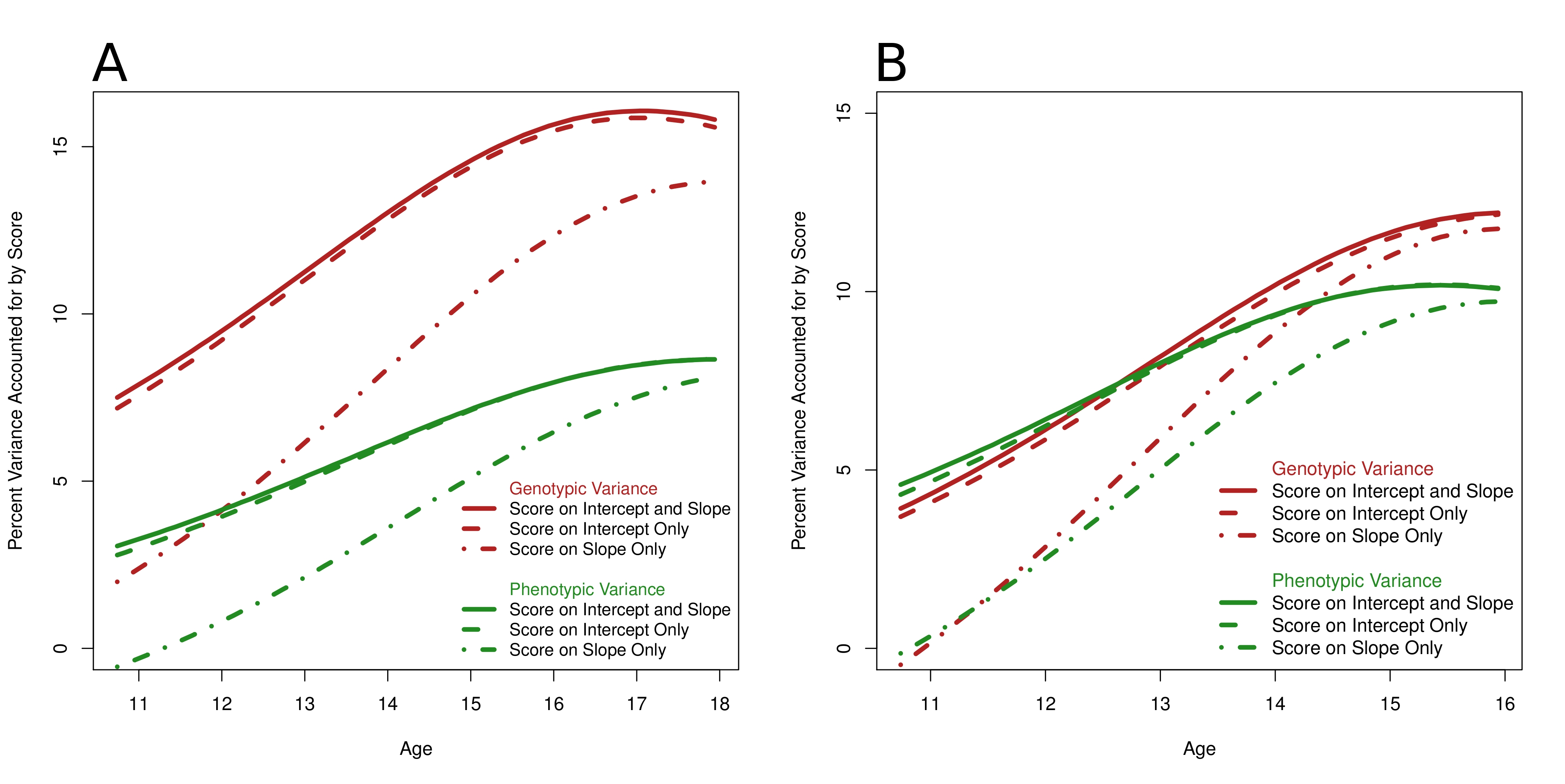

Supplement: Figure S2 — (a) Refers to males; (b) to females. All other aspects are described in the caption to Figure 4 in the main text. (TIF) [file pgen.1002413.s002.tif]

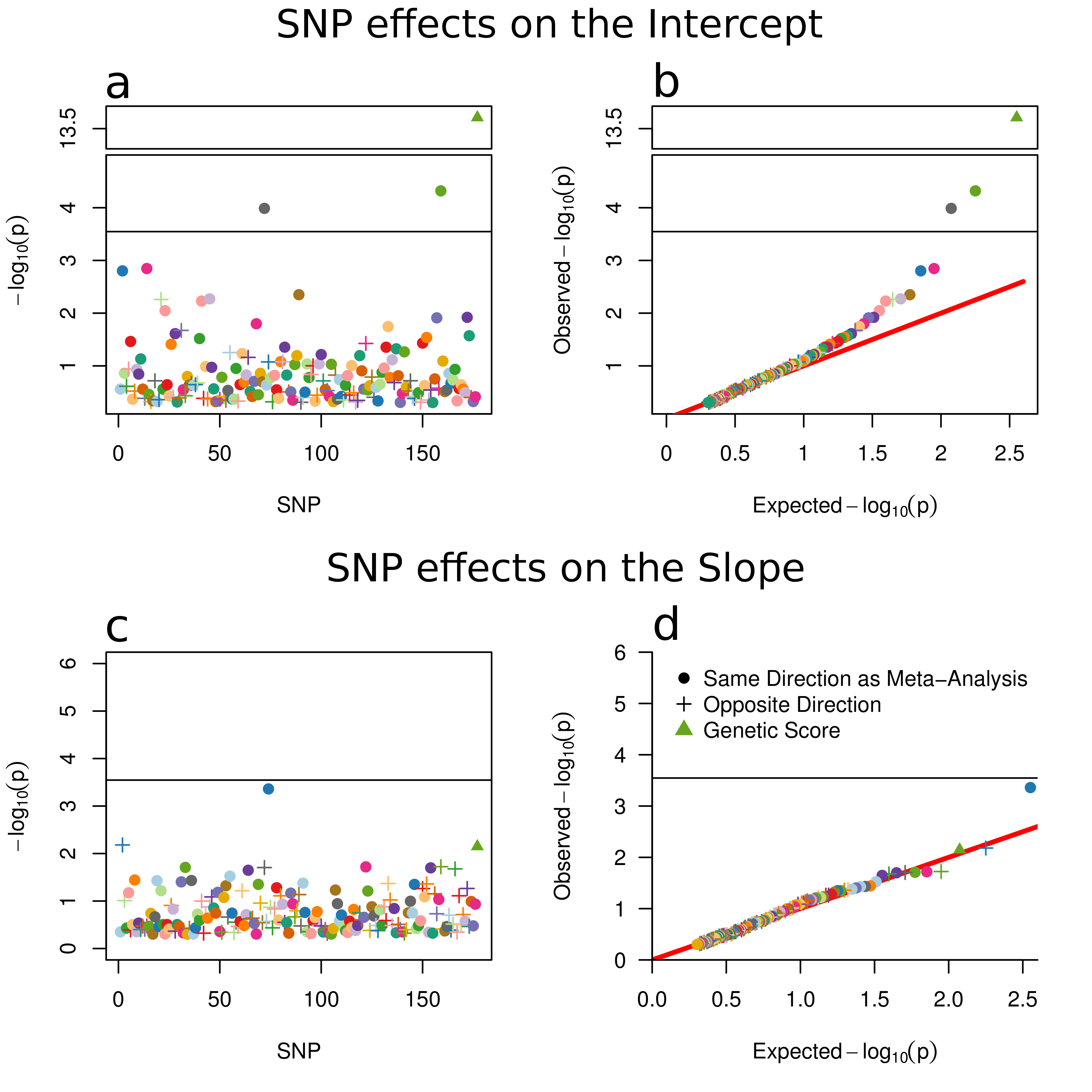

Supplement: Figure S3 — SNP and Score Effects on the Puberty-Corrected Growth Model Intercept and Slope. Univariate plot (a) and QQ plot (b) of SNP effects on intercept. Univariate plot (c) and QQ plot (d) of SNP effects on the slope. All symbols are described in the caption for Figure 2. The SNP effects are relatively stronger for age-11 height (panels (a) and (b)) than for pubertal growth (panels (c) and (d)). The genetic score is highly significant for age-11 height but not pubertal growth. The single Bonferroni-significant effect for the slope (highest blue dot in panels (c) and (d)) is the only SNP, of all 180 identified in the Allen et al. [7] meta-analysis, that has been linked to pubertal growth in height [26]. All p-values are also listed in Table S1. Each mark is colored to allow easy cross-referencing between panels, and also between Figure 2 and Figure 3. (TIF) [file pgen.1002413.s003.tif]
