# Supplementary material for: An Assessment of the Individual and Collective Effects of Variants on Height Using Twins and a Developmentally Informative Study Design
Source: PLoS Genet. 2011 Dec 8;7(12):e1002413. doi: 10.1371/journal.pgen.1002413 (PMC3234218; doi:10.1371/journal.pgen.1002413)
Supplement: Table S2 — Regression Correlation Coefficient Matrix. These are Spearman rank order correlations between all 176 regression coefficients computed for each height phenotype under study. Confidence intervals were computed by bootstrap with 2000 pseudo-replications. “Meta-Analysis” refers to sex-combined regression coefficients from the GIANT Consortium meta-analysis [7]. “Age-11 Height” and “Pubertal Growth” are the SNP effects on the growth model intercept and slope, respectively. “Adult Height” is the full adult height analysis described in the present report. All values are statistically significant, indicating a general trend for the SNP effects regardless of height phenotype analyzed. The full scatterplot matrix is given in Figure S1. (DOC) [file pgen.1002413.s005.doc]

|  | Meta-Analysis | Age-11 Height | Pubertal Growth | Adult Height |
| --- | --- | --- | --- | --- |
| Meta-Analysis | 1 |  |  |  |
| Age-11 Height | .45 (.32, .58) | 1 |  |  |
| Pubertal Growth | .17 (.001, .32) | -.54 (-.65, -.42) | 1 |  |
| Adult Height | .82 (.78, .86) | .50 (.37, .62) | .20 (.04, .34) | 1 |
